# Serum S100B Levels Can Predict Computed Tomography Findings in Paediatric Patients with Mild Head Injury

**DOI:** 10.1155/2018/6954045

**Published:** 2018-04-23

**Authors:** Fatos M. Kelmendi, Arsim A. Morina, Agon Y. Mekaj, Afrim Blyta, Ridvan Alimehmeti, Shefki Dragusha, Feti Ahmeti, Qamile Morina, Afrim Kotori

**Affiliations:** ^1^Clinic of Neurosurgery, University Clinical Center of Kosovo, 10000 Pristina, Kosovo; ^2^Clinic of Neurology, University Clinical Center of Kosovo, 10000 Pristina, Kosovo; ^3^Service of Neurosurgery, University Hospital Center “Mother Theresa”, 370 Dibra Street, Tirana, Albania; ^4^Clinic of Anesthesiology, University Clinical Center of Kosovo, 10000 Pristina, Kosovo; ^5^Clinic of Medical Biochemistry, University Clinical Center of Kosovo, 10000 Pristina, Kosovo

## Abstract

**Introduction:**

Traumatic brain injuries (TBIs) are very common in paediatric populations, in which they are also a leading cause of death. Computed tomography (CT) overuse in these populations results in ionization radiation exposure, which can lead to lethal malignancies. The aims of this study were to investigate the accuracy of serum S100B levels with respect to the detection of cranial injury in children with mild TBI and to determine whether decisions regarding the performance of CT can be made based on biomarker levels alone.

**Materials and Methods:**

This was a single-center prospective cohort study that was carried out from December 2016 to December 2017. A total of 80 children with mild TBI who met the inclusion criteria were included in the study. The patients were between 2 and 16 years of age. We determined S100B protein levels and performed head CTs in all the patients.

**Results:**

Patients with cranial injury, as detected by CT, had higher S100B protein levels than those without cranial injury (*p* < 0.0001). We found that patients with cranial injury (head CT+) had higher mean S100B protein levels (0.527 *μ*g L^−1^, 95% confidence interval (CI) 0.447–0.607 *μ*g L^−1^) than did patients without cranial injury (head CT−) (0.145 *μ*g L^−1^, 95% CI 0.138–0.152 *μ*g L^−1^). Receiver operating characteristic (ROC) curve analysis clearly showed that S100B protein levels differed between patients with and without cranial injury at 3 hours after TBI (AUC = 0.893, 95% CI 0.786–0.987, *p* = 0.0001).

**Conclusion:**

Serum S100B levels cannot replace clinical examinations or CT as tools for identifying paediatric patients with mild head injury; however, serum S100B levels can be used to identify low-risk patients to prevent such patients from being exposed to radiation unnecessarily.

## 1. Introduction

Traumatic brain injury (TBI) is a very frequent cause of emergency department visits in paediatric populations. TBI is also one of the leading causes of death and morbidity in children [1]. Communication difficulties sometimes obstruct the acquisition of a detailed injury history and the identification of signs of TBI in an accurate and timely manner in paediatric patients. The vast majority of children with mild TBI, who by definition present with a Glasgow Coma Scale (GCS) score of 13–15, have no intracranial injury (ICI). Lesions are seen in 3–7% of children who undergo computed tomography (CT). Moreover, only 0.1–0.6% of patients need neurosurgical intervention [2, 3]. These patients are notoriously difficult to manage, as they have a low but nonnegligible risk of intracranial complications, which may be life threatening [4]. CT overuse has become a controversial issue because ionizing radiation exposure can lead to lethal malignancies. The incidence of malignancy may be as high as 1 in 1,000 head CTs. In a recent survey of radiologists and emergency-room physicians, approximately 75% of the entire group significantly underestimated the radiation dose from a CT scan, and 53% of radiologists and 91% of emergency-room physicians did not believe that CT scans increased the lifetime risk of cancer [5]. Younger children are more susceptible to developing malignancy than are other patients [6, 7]. The dose delivered to the brain of an infant during CT of the skull is approximately 120 mGy. A decrease in high school attendance was observed in all socioeconomic groups at radiation doses greater than 100 mGy compared with that at the lowest dose of 1–20 mGy [8]. The signs and symptoms of some types of TBI (e.g., epidural haematoma, subdural haematoma, or brain contusion) may manifest themselves after a delay, even in patients whose neurologic examinations and initial head CTs were negative. Thus, subjecting all patients with mild TBI to CT to exclude cranial injury is inefficient. Clinicians have developed several clinical criteria to reduce the numbers of unnecessary CTs [2, 9, 10]. In the paediatric head injury population, the decision regarding whether to perform CT depends mostly on the experience of the treating emergency physician. Physician experience is the key factor determining the management of paediatric patients with head injuries, although current guidelines are available for the treatment of mild TBI in children [11, 12]. These guidelines do not advocate the use of S100B in paediatric patients, as the number of paediatric studies is insufficient. The above findings underscore the need to design a diagnostic tool that displays good diagnostic performance; is easy to use, minimally invasive, and cost effective; and predicts outcomes in a timely and efficient manner [13]. Over the past 15 years, clinicians have proposed using S100B protein levels as an initial screening tool in patients with suspected brain damage [14]. Although extracranial sources of S100B, such as fat, muscle, and bone marrow, may contribute to S100B levels, minor peripheral injuries do not appear to lead to significant rises in S100B levels [15]. S100B has been shown to be highly sensitive with respect to the detection of brain damage and may be more sensitive for the detection of TBI than CT. Thus, S100B levels may have a high negative predictive value (NPV) in paediatric patients with mild head injury [16] as an evolving injury may not be apparent on CT at the time of initial presentation. Serum S100B protein can be used to determine which patients require an emergent CT, which patients can be observed in the emergency department, which patients can be discharged without further observation or testing, and which patients should be admitted to the hospital so that they can be observed more closely in a neurosurgical ward. In patients with high levels of S100B and an isolated head injury, head CT should be performed immediately, and the patient should be closely monitored in a neurosurgical ward. Patients with normal levels of S100B can be monitored for up to four hours in the emergency department and then discharged and sent home. Patients admitted to the emergency department after three hours from the time of injury and patients with multiple injuries were not included in the study because the S100B values were not accurate. A few studies have evaluated the usefulness of S100B as a tool for reducing the numbers of CTs that are performed in children with mild TBI [17, 18]. The results of several studies from separate research groups and a meta-analysis indicate that S100B has shown NPV over 99% and close to 100% for intracranial complications and neurosurgical lesions after mild TBI, respectively [16].

Age may also influence S100B levels. S100B levels in children aged 1 and 2 years are significantly higher than those in children aged 3–14 years [19, 20].

The aims of this study were to assess the accuracy with which serum S100B levels can detect cranial injury in children with mild TBI and to determine whether the decision regarding the performance of a CT can be made using biomarkers alone.

## 2. Materials and Methods

### 2.1. Study Design

This was a single-center prospective cohort study that was carried out from December 2016 to December 2017. The investigation was conducted in compliance with the International Conference on Harmonization Guidelines for Good Clinical Practice [21] and the Declaration of Helsinki [22]. The study was approved by the Ethics Committees of the Medical Faculty/University of Pristina, reference number 792, and the University Clinical Center of Kosovo, reference number 3624/2. The study is registered in the ClinicalTrials.gov data base. The registration number is NCT02988102.

### 2.2. Settings and Participant Selection

The study was conducted in the emergency department and the neurosurgery clinic. The study site is a tertiary neurosurgical center in a region with more than 1.7 million inhabitants. Children with head trauma alone who were between 2 and 16 years of age were included in the study. We aimed to restrict the study to paediatric patients with isolated minor head injuries because other traumatic injuries have been reported to increase serum S100B levels, a phenomenon that may lead to false-positive results. Informed consent was obtained from the parents or a legal representative (either immediately before the child was included in the study or immediately after the parent or representative had agreed to allow the child to participate) in the neurosurgery clinic.

Children who were admitted to the hospital more than three hours after trauma, children with a history of syncope or seizure before the head trauma, children with Down syndrome (S100B is overexpressed in such patients) [23], children who had previously undergone a neurosurgical procedure, children with multiple injuries (involving the chest, abdomen, extremities, or pelvis), children with renal or liver disease, children with a history of a severe neurologic or psychiatric disorder, children suffering from cancer, and children who either had a history of an inherited coagulopathy or had received anticoagulant therapy were excluded from the study. We did not perform additional laboratory tests to determine each patient's international normalized ratio, platelet count, platelet function, or prothrombin time because there is often insufficient time for the performance of such tests in a busy emergency department. Serum S100B protein has a short half-life; thus, patients whose blood samples were drawn more than 3 hours after head trauma were excluded from the analysis. Collectively, the net amount of S100B in serum was considered a reflection of the combined rates of influx and elimination. As the serum half-life of S100B is short, prolonged elevation of S100B indicates ongoing influx [24]. According to the guidelines proposed by the European Federation of Neurological Societies (EFNS), the patients were diagnosed with mild TBI if they presented with a GCS score of 13–15, loss of consciousness (LOC) lasting <30 minutes, and posttraumatic amnesia (PTA) lasting <1 hour [25]. Comprehensive clinical examinations were performed in all patients.

### 2.3. Blood Sampling and S100B Concentration Measurement

Blood samples were obtained from each patient via a cubital vein at 3 hours after head injury. The blood samples were processed to separate the serum from the plasma, and then the serum was deep-frozen at −20°C (−4°F) until analyzed with an electrochemiluminescence immunoassay kit (Elecsys S100; Roche Diagnostics, Mannheim, Germany). According to the manufacturer's instructions, the test system requires an 18-minute probe volume of at least 20 *μ*L of serum. The lower limit of detection is 0.005 *μ*g/L, and concentrations of up to 39 *μ*g/L can be measured without dilution. The results are reported in micrograms per litre and are rounded to three decimal places. S100B levels had no effect on clinical decisions or patient management in our study.

### 2.4. Radiographic Examination

An emergency head CT was performed in the emergency department in accordance with a protocol requiring the acquisition of 5-mm-thick slices from the skull base to the vertex and 2-mm-thick slices throughout the skull base to generate images. The CT was usually performed within 30 minutes after the patient was first examined by an emergency physician. A venous blood sample was drawn prior to every CT. The CT examination involved the acquisition of parenchymal and bone window images. All head CTs were reviewed for signs of TBI by a radiologist blinded to the patients' clinical signs and S100B levels. Patients found to have any signs of trauma-related cerebral lesions (skull-cap fracture, skull-base fracture, or both; epidural haematoma; subdural haematoma; traumatic subarachnoid bleeding; cerebral haematoma; brain contusion; or pneumocephalus) were considered to have a positive head CT. The patients were classified according to the number of injuries rather than the size of the injury. Many of the patients had multiple injuries, but these injuries affected only a small volume of brain tissue (always considering that one injury can damage a large area of brain tissue). We did not perform plain radiographs of the skull because negative radiographs do not exclude intracranial bleeding. Additionally, we did not perform an MRI.

### 2.5. Statistical Analysis

The data were analyzed using SPSS Statistics 22.0 software (IBM, Chicago, IL, USA) and are presented as the means and standard deviations [SDs] or as the medians and interquartile ranges. A 95% confidence interval (CI) was calculated for all values using the normal approximation method. Qualitative variables were tested using a *X*^2^-test or Fisher's exact test, and quantitative variables were tested using Student's *t*-test, the Mann–Whitney test *U*, and ANOVA followed by Dunn's post hoc test for multiple comparisons. Spearman correlation and receiver operating characteristic (ROC) curve analyses were also performed. The Kruskal–Wallis test was used to evaluate S100B values by age group. *p* < 0.05 was considered significant.

## 3. Results

A total of 80 patients with mild TBI met the inclusion criteria for this study. Forty-six patients were male (57.5%), and 34 patients were female (42.5%). The mean age was 9.1 years (SD ± 3.8 years). The patients were divided into the following two groups: (a) a negative CT group (CT−), which included patients without any signs of cranial injury on CT; and (b) a positive CT group (CT+), which included patients with at least one trauma-related lesion on CT. The demographic and clinical characteristics of the patients with mild head injury, as demonstrated by CT, are shown in Table 1.

The mean S100B level in our series was 0.398 *μ*g L^−1^ (SD ± 0.298 *μ*g L^−1^), and the 95% CI ranged from 0.332 to 0.465 *μ*g L^−1^. A total of 53 patients (66.3%) had cranial lesions.

Patients with cranial injury, as demonstrated by CT, had higher S100B protein levels than those without cranial injury (*p* < 0.0001). The mean serum S100B protein level in patients without cranial injury (head CT−) was 0.145 *μ*g L^−1^ (95% CI 0.138–0.152 *μ*g L^−1^), while the mean serum S100B protein level in patients with cranial injury (head CT+) was 0.527 *μ*g L^−1^ (95% CI 0.447–0.607 *μ*g L^−1^).

**Figure 1:**
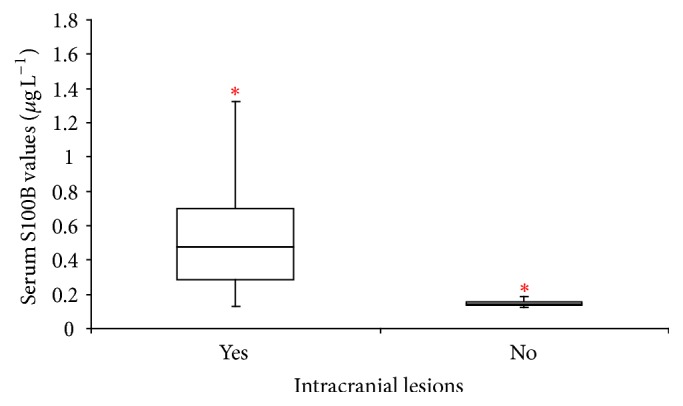
Box-and-whisker plots of serum S100B levels (*μ*g/L) in patients with and without cranial injury. ^*∗*^An extreme value within group.

As shown in Figure 1, the median S100B level in patients without cranial injury was 0.141 *μ*g L^−1^, and the median S100B level in patients with cranial injury was 0.478 *μ*g L^−1^.

**Figure 2:**
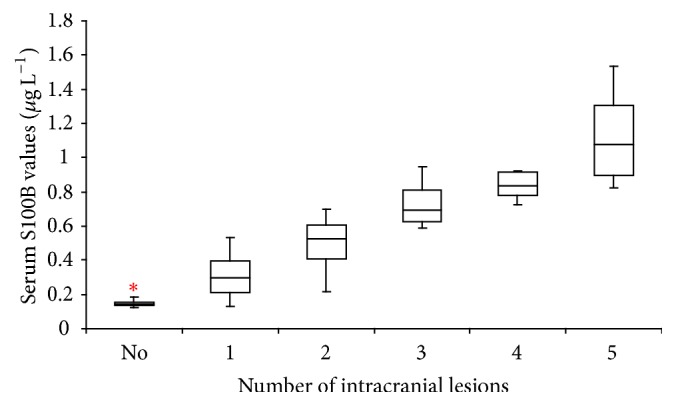
Box-and-whisker plots of serum S100B levels (*μ*g/L) according to the number of cranial injuries observed on CT: no cranial injury (No), one cranial injury (1), two cranial injuries (2), three cranial injuries (3), four cranial injuries (4), and five cranial injuries (5). ^*∗*^An extreme value within group.

The patients who suffered multiple cranial injuries were grouped based on their CT findings. The mean S100B values were 0.145, 0.310, 0.496, 0.733, 0.834, and 1.127 *μ*g L^−1^ in patients without cranial injury, patients with one cranial injury, and patients with two, three, four, and five cranial injuries, respectively, as shown in Table 2. We noted a positive correlation between cranial injury number and S100B levels (Spearman's rho = 0.924, *p* ≤ 0.001) (see Figure 2).

**Figure 3:**
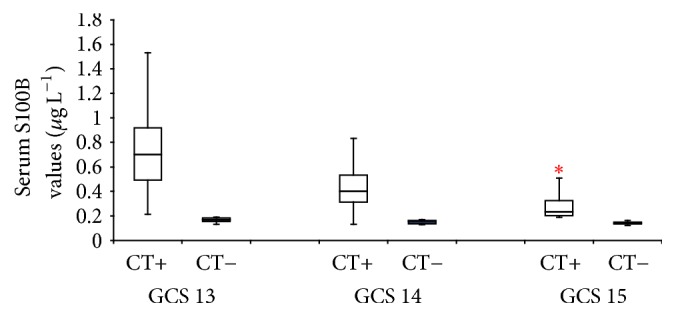
S100B serum concentrations according to patient GCS scores upon admission to the emergency department. This graph groups the median (and interquartile range) serum S100B concentrations according to the patients' initial GCS scores upon their admission to the emergency department. The black squares indicate the concentrations of the patients without cranial injury (CT−), and the open squares indicate the concentrations of the patients with cranial injury (CT+). S100B concentrations were significantly increased in the patients with cranial injury (CT+) compared with those in the patients without cranial injury (CT−) in each GCS group (^*∗*^*p* < 0.001 in the *U* test, CT+ versus CT−). Among the patients with cranial injury (CT+), S100B concentrations in the patients with GCS scores of 15 were significantly lower than those in the patients with GCS scores of 13 or 14 (*p* < 0.05 in ANOVA followed by Dunn's post hoc test for multiple comparisons). Among the patients without cranial injury (CT−), no significant differences were observed in S100B concentrations in the patients with GCS scores of 13, 14, or 15.

As shown in Figure 3, the S100B concentrations (median [interquartile range]) in the GCS 13 group were significantly different between head CT+ (0.701 *μ*g L^−1^ [0.212–1.532 *μ*g L^−1^]) and CT− (0.170 *μ*g L^−1^ [0.132–0.190 *μ*g L^−1^]) groups (*U* test, *p* = 0.0001). Significant differences were also observed in the GCS 14 between CT− (0.159 *μ*g L^−1^ [0.128–0.170 *μ*g L^−1^]) and CT+ (0.401 *μ*g L^−1^ [0.132–0.832 *μ*g L^−1^]) groups (*t*-test, *p* = 0.004) and GCS 15 between CT− (0.140 *μ*g L^−1^ [0.121–0.162 *μ*g L^−1^]) and CT+ (0.232 *μ*g L^−1^ [0.187–0.601 *μ*g L^−1^]) groups (*U* test, *p* < 0.0001). The concentrations of S100B in the patients without cranial injury (CT−) in the GCS 13, 14, and 15 groups were not significantly different by ANOVA on ranks calculation, whereas the S100B concentrations in the patients with cranial injury (CT+) in the GCS 15 group were lower than those in the GCS 13 or 14 groups, as determined by ANOVA on ranks.

As shown in Table 3, the serum S100B value increased with age. The median S100B value was 0.308 *μ*g L^−1^ (SD ± 0.254 *μ*g L^−1^) at <5 years of age, 0.338 *μ*g L^−1^ (SD ± 0.253 *μ*g L^−1^) at 5–9 years of age, and 0.476 *μ*g L^−1^ (SD ± 0.329 *μ*g L^−1^) at 10+ years of age by the Kruskal–Wallis test. However, we did not observe statistically significant differences among the groups (*p* = 0.084).

**Figure 4:**
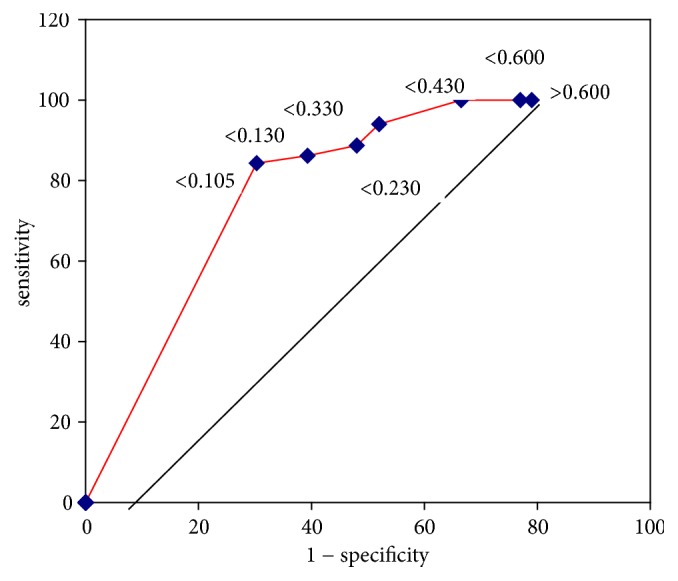
ROC analysis comparing sensitivity and specificity 3 h after TBI.

The ROC analysis in Figure 4 shows that S100B levels differed significantly between the patients with and without cranial injury at 3 hours after TBI (AUC = 0.893, 95% CI 0.786–0.987, *p* = 0.0001).

## 4. Discussion

The first report concerning the use of serum S100B as a biomarker for mild head injury was published in 1995 [26]. Numerous reports and a meta-analysis on the ability of S100B to safely reduce the number of CTs performed in patients with mild head injury have since increased the body of evidence supporting its clinical use [27]. In many cases, S100B concentrations can be used to predict whether intracranial pathologic findings that are visible on head CT are present. Clinicians must weigh the small chance of missing an intracranial injury against the risks and cost associated with scanning paediatric patients when making decisions regarding the performance of a head CT. Such decisions are also made based on whether the performance of the CT involves the sedation of the patient or the transfer of the patient to another facility. All known sets of guidelines for the management of mild head injury state that a CT is mandatory in patients with a GCS score of 13, and most sets of guidelines recommend the performance of a CT in patients with a history of LOC and GCS scores of 14 to 15 [28]. Our analysis showed that S100B measurements can predict the likelihood of head CT abnormalities in paediatric patients with GCS scores of 14 to 15. Our prospective study showed that the S100B protein is useful for detecting patients at risk for intracranial injury. We assessed the ability of initial serum S100B concentrations to predict the likelihood of positive CT findings in a sample of 80 paediatric patients with minor head injuries. Our results are consistent with those of another large-scale study performed by Biberthaler et al. [27]. These authors studied a sample of 1,309 patients with minor head injuries and found that serum S100B levels have an NPV of 99.7% (95% CI 99% to 100%) and can enable the avoidance of CT in 28% of patients with minor head injuries. The median serum S100B level in patients with a negative CT was 0.16 *μ*g L^−1^ in the study by Biberthaler et al. In our study, the median S100B level was 0.14 *μ*g L^−1^. We noted a positive correlation between cranial injury numbers and S100B levels (Spearman's rho = 0.924, *p* ≤ 0.001). The 0.105-*μ*g L^−1^ cut-off described in the literature [27, 29] was shown to be an excellent screening tool, as it displayed a sensitivity of 100% and a specificity of 26.56% for the detection of cranial injury (*p* = 0.022). However, the data showed that using a higher cut-off, namely, 0.130 *μ*g L^−1^, increased the specificity to 32.81%. Analysis showed that, in the group of patients with positive head CTs, serum S100B concentrations differed significantly between patients with GCS scores of 13 or 14 and those with GCS scores of 15. However, in the group of patients with negative head CTs, serum S100B scores did not differ significantly among patients with GCS scores of 13, 14, or 15. In our study, we found that epidural haematomas do not cause increases in S100B levels unless they are associated with another brain parenchymal injury. There is a concern that epidural haematomas might not produce increased S100B levels, because these lesions might not always be associated with extensive brain parenchymal injury. In the present study, the two patients with isolated epidural hematoma had S100B levels of 0.11 and 0.21 *μ*g L^−1^, respectively [29]. Only patients who presented to the emergency department within 3 hours of a traumatic event were included in our study, as previous animal and human studies have reported that the half-life of the relatively small S100B protein (21 kDa) is estimated to range from 25 to 120 minutes [26, 30, 31]. We attempted to exclude patients with small intracranial lesions who presented to the emergency department after a delay because we were concerned that these patients' serum S100B protein concentrations would fall below the abovementioned cut-off. Our data show that although S100B is rapidly cleared from the serum, the concentrations of the protein are unlikely to fall to a normal level within 3 hours in paediatric patients with prognostically mild injuries. Using S100B as the only identifier of cranial injury would have enabled the avoidance of 27 (33.75%) head CT scans, but 66.25% of the lesions requiring closer monitoring would have been missed. Therefore, the decision regarding whether to perform head CT should not be determined based on the level of this biomarker alone. Clinicians have developed several validated clinical symptom-related criteria to reduce the frequencies of unnecessary CT scans and radiation-induced malignancies [9]. The strongest criteria were described in a study by Kuppermann et al. who suggested performing CT in all children with a GCS score <15 or signs of altered mental status or basilar skull fracture. These authors also suggested that the treating physician should make the decision regarding the performance of head CT in a child with a history of LOC, vomiting, an injury with an unusual mechanism, or severe headache [9]. The authors stated that each physician should base this decision on his or her experience. S100B levels may help physicians avoid ordering unnecessary head CT scans in these cases. CTs are among the most expensive tests ordered in our emergency department. Our study showed that S100B levels are helpful for ruling out TBI after minor head injuries and may therefore reduce the frequency with which head CT scans are performed in the emergency department. Our findings are consistent with those of Papa et al. [32] and Berger et al. [33]. Reducing the frequency with which unnecessary head CT scans are performed may decrease patient exposure to radiation, as well as the time spent in the emergency department, as patients often endure 4-hour delays before undergoing CTs. The S100B test does not require costly equipment and is inexpensive.

## 5. Conclusion

Serum S100B levels cannot replace clinical examinations or CT to identify paediatric patients with mild head injuries; however, they may be used to identify low-risk patients to prevent their unnecessary exposure to radiation. Including recommendations for measurement of S100B levels in the guidelines for the management of mild head injury may prevent unnecessary CT scans, which is currently recommended for patients with mild head injury, and thus reduce radiation exposure in the paediatric population and save precious healthcare resources. Measuring S100B protein levels may improve emergency patient care by reducing unnecessary tests and lengths of stay in busy emergency departments. Our findings clearly indicate that S100B protein levels are an accurate tool for ruling out intracerebral injuries.

## Figures and Tables

**Table 1:** Demographic and clinical characteristics of the patients with mild head injury according to CT.

	CT+ *n* (%)	CT− *n* (%)	Total *n* (%)	*p* value
Total	53 (100.0)	27 (100.0)	80 (100.0)	
*Age (year)*				
Mean ± SD	9.6 ± 3.5	8.0 ± 4.0	9.1 ± 3.8	*p* = 0.103
*Gender*				
Male	31 (58.5)	15 (55.6)	46 (57.5)	*p* = 0.990
Female	22 (41.5)	12 (44.4)	34 (42.5)	
*Mechanism of trauma*				
Traffic	17 (32.1)	6 (22.2)	23 (28.8)	*p* = 0.940
Sports injury	10 (18.9)	3 (11.1)	13 (16.3)	
Fall	12 (22.6)	7 (25.9)	19 (23.8)	
Other	15 (28.3)	10 (37.0)	25 (31.3)	
*Symptoms, findings*				
GCS 15	8 (15.1)	17 (63.0)	25 (31.3)	*p* = 0.001
GCS 14	21 (39.6)	5 (18.5)	26 (32.5)	
GCS 13	23 (43.4)	4 (14.8)	27 (33.8)	

Amnesia	19 (35.8)	4 (14.8)	23 (28.8)	*p* = 0.067
LOC	36 (67.9)	8 (29.6)	44 (55.0)	*p* = 0.001
Nausea	23 (43.4)	16 (59.3)	39 (48.8)	*p* = 0.238
Vomiting	36 (67.9)	10 (37.0)	46 (57.5)	*p* = 0.015
Headache	53 (100.0)	22 (81.5)	75 (93.8)	*p* = 0.003
Dizziness	17 (32.1)	13 (48.1)	30 (37.5)	*p* = 0.222

SD: standard deviation; GCS: Glasgow Coma Scale; LOC: loss of consciousness prior to hospital admission.

**Table 2:** Number of intracranial lesions detected on CT and S100B levels.

Serum S100B levels (*μ*g L^−1^)	Number of intracranial lesions					
	No	1	2	3	4	5
*N*	27	24	12	8	5	4
Mean	0.145	0.310	0.496	0.733	0.834	1.127
SD	0.018	0.110	0.156	0.136	0.087	0.321
Min	0.121	0.132	0.214	0.59	0.721	0.821
Max	0.19	0.531	0.701	0.945	0.923	1.532

A CT scan was considered positive if at least one trauma-relevant lesion was detected (skull-cap fracture, skull-base fracture, or both; epidural haematoma; subdural haematoma; traumatic subarachnoid bleeding; cerebral haematoma; brain contusion; or pneumocephalus).

**Table 3:** S100B levels by age group.

Serum S100B values (*μ*g L^−1^)	Age group (years)		
	<5	5–9	10+
*n*	13	29	38
Mean	0.308	0.338	0.476
SD	0.254	0.253	0.329
Min	0.127	0.121	0.121
Max	0.923	1.230	1.532

*p* value	*p* = 0.084		

Although serum S100B levels increased with age, the Kruskal–Wallis test did not identify statistically significant differences among the groups (*p* = 0.084) with median S100B levels of 0.308 *μ*g L^−1^ (SD ± 0.254 *μ*g L^−1^), 0.338 *μ*g L^−1^ (SD ± 0.253 *μ*g L^−1^), and 0.476 *μ*g L^−1^ (SD ± 0.329 *μ*g L^−1^) in children <5 years old, 5–9 years old, and 10+ years old, respectively.
